# Broadband Frequency Scanning Spoof Surface Plasmon Polariton Design with Highly Confined Endfire Radiations

**DOI:** 10.1038/s41598-019-56720-4

**Published:** 2020-01-10

**Authors:** Abhishek Kandwal, Jingzhen Li, Tobore Igbe, Yuhang Liu, Sinan Li, Lei Wang, Yang Hao, Zedong Nie

**Affiliations:** 1 Shenzhen Institutes of Advanced Technology, Chinese Academy of Sciences, Shenzhen, China; 2 School of Electronic Engineering and Computer Science, Queen Mary University of London, London, UK

**Keywords:** Electrical and electronic engineering, Applied physics

## Abstract

This paper proposes a compact broadband frequency scanning spoof surface plasmon polariton (SSPP) based design for efficient endfire radiations with high field confinement. Through the dispersion engineering, highly confined field distribution has been obtained in the operating frequency region. The proposed SSPP antenna has achieved a continuous through endfire scanning in the X-band at other operating frequencies which is in general difficult to obtain for SSPP based antennas. In the proposed design methodology, the swore-shaped surface plasmon antenna has both edges corrugated with an array of rectangular grooves which effectively confines the electromagnetic field into a slow travelling wave. The surface impedances along both edges were engineered to be different at operating frequencies as to force the surface current to preferentially flow along either edge of the antenna to a different extent. The design with overall dimensions of (55 × 30) *mm*^2^ has achieved a broadband of 4 GHz with high peak measured gain of 9.8 dBi and peak efficiency of about 95 percent in the X-band. The antenna has been further tested experimentally for scanning application of target location also.

## Introduction

Surface plasmons are the quanta of surface-charge-density oscillations, but the same terminology is commonly used for collective oscillations in the electron density at the surface of a metal. Mimicking the features of surface plasmons in the microwave frequencies has led to evolution of term “spoof”. In the recent years, the research on spoof surface plasmon polariton (SSPP) based designs such as meta-materials, antennas has gained lot of attention due to their attractive features such as high field confinement at the metal-dielectric interface which makes them very suitable for the variety of applications including spectroscopy and microwave imaging^1–3^. Surface plasmon polaritons propagate along the interface between metal and dielectric. These decay exponentially in the transverse direction in the optical regime. The SSPP antennas in general radiates in the broadside direction and are able to provide frequency scanning through broadside direction. However very few papers have been published till now on SSPP endfire radiating antennas due to specific structure requirements. As a topic of interest, SSPP based endfire antennas have been reported in the recent years but only few of them have been able to provide through endfire frequency scanning along with endfire radiation due to their narrow band performance. Many of the endfire antenna designs were previously based on the well known Vivaldi methodology which was published at the Ninth European Microwave Conference (IEEE) in 1979^4,5^. The concept of spoof surface plasmon based designs have led to the rise of new class of endfire radiating antennas based on surface wave theory. Different kinds of SSPP based structures have been proposed till now and the study shows that proposed devices can be used in applications from microwave, millimeter waves to terahertz frequencies^6–12^.

In^13^, a spoof surface plasmon design has been proposed using metamaterials. This antenna in general outperforms many other counterparts due to the use of the I-shaped metamaterial elements. However, the endfire radiation has been achieved at the expense of an increase in the overall effective aperture. Tian *et al*. recently proposed a low-profile bidirectional endfire antenna based on spoof surface plasmon polaritons^14^. But a linear continuous beam scanning has not been achieved in SSPP based endfire antennas till now especially without the use of active components^15,16^. In^15^, the beam scan obtained is just 6 degrees in the endfire direction using diodes.

In^17^, a uniplanar microstrip antenna has been proposed for endfire radiation. The design has obtained a gain of 8.2 dBi with a narrow bandwidth of 100 MHz. The overall dimensions are (58 mm × 43 mm). The antenna is compact but the bandwidth is quiet narrow. In^18,19^, a high gain of about 12 – 13 dBi has been achieved at the expense of large overall size for achieving endfire radiations. Dimensions of (444 mm × 132 mm) and (406 mm × 80 mm) have been used by these designs respectively. The impedance bandwidth is around 3.5 GHz in^17^ and around 1 GHz in^19^. In^19^, the main beam has been shifting non-linearly at different angles in the endfire direction thereby producing non-linear scanning. In^20^, an efficient method to radiate surface plasmon polaritons (SPPs) directly using metallic corrugated strip with gradient grooves and flaring structure has been proposed. Although the design has achieved high gain but at the expense of large overall size and flaring structure. The authors own previous work have proposed a methodology to achieve continuous broadside scanning using a Goubau line leaky wave design^21^. In another work^22^, a travelling wave SSP based antenna has been proposed. The design was based on U-shaped periodic unit cells with large overall size and just one sided corrugations. It was also not able to achieve exactly endfire radiations and no frequency scanning feature was observed.

In this work, we proposed a fundamentally new design methodology with asymmetric upper and lower corrugations and H-shaped periodic unit cells for designing a compact broadband SSPP-based antenna to obtain exact endfire radiations with frequency scanning capability. It is to be noted carefully that the proposed paper focusses only on spoof surface plasmon based endfire radiation class of antennas. The proposed design methodology has successfully yielded a substantially improved version in all aspects than most of the published SSPP endfire antennas. More importantly, the most essential aspect of the proposed SSPP antenna from the proposed design methodology is indeed the exact endfire radiation with frequency scanning capability, which includes the abilities to exhibit through endfire scanning across X-band. As the design has a large operating band, the frequencies other than the one for exactly endfire radiations, can be used for scanning applications if a continuous beam scanning is obtained. This will allow one design to perform multi-function for applications that require just endfire radiations or that require frequency scanning also. The proposed SSPP design can be used for applications where scanning is required such as imaging applications. One such application is target location and detection. We have performed a prefatory experiment here to show the benefit of the design. The main features of the proposed SSPP design include: (1) Efficient endfire radiation (2) Compact size (3) Through endfire scanning representing multifunction (4) Scanning application.

## Principle and Dispersion Analysis of Spoof Surface Plasmon Design

**Figure 1:**
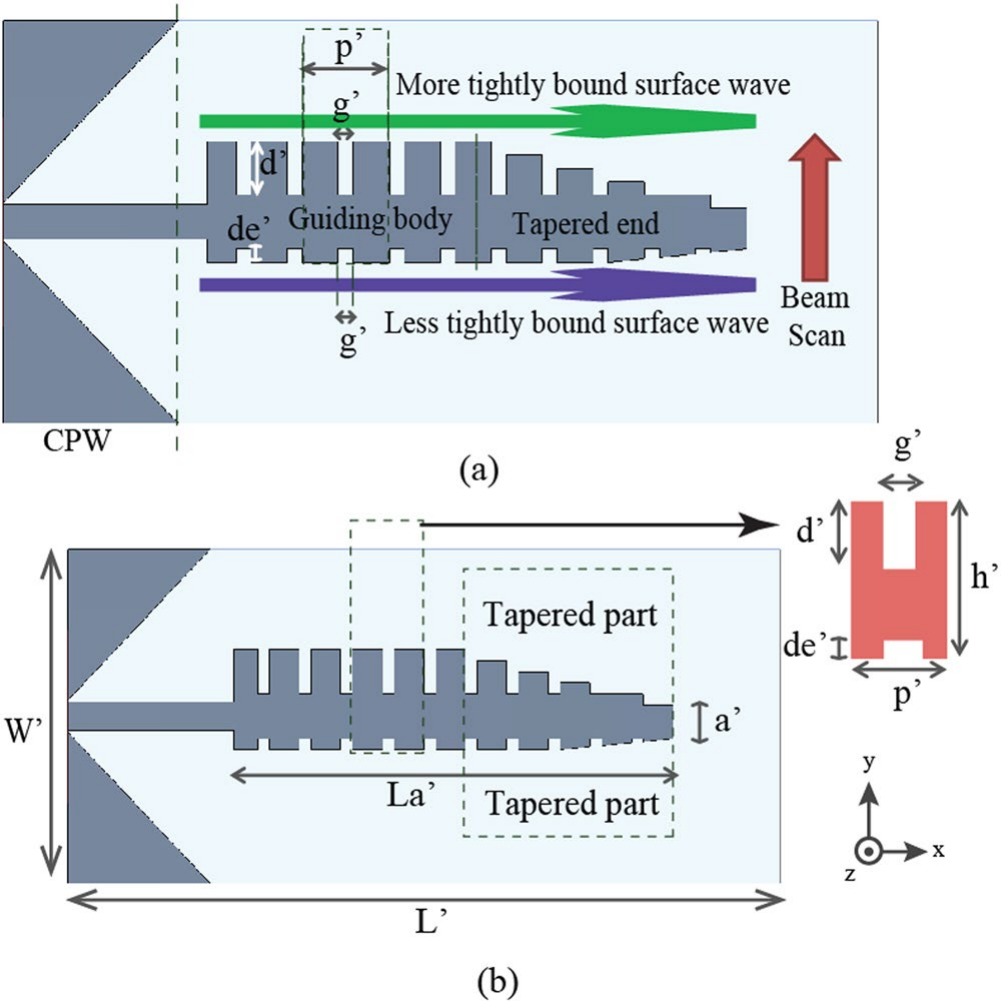
Configuration of the SSPP antenna (**a**) Concept (**b**) Design.

Figure 1 shows the topology of the proposed SSPP based antenna. The antenna can be divided into three sections, including the CPW feeding section, the main body of the antenna and the tapered impedance end. The guiding body of antenna is a Spoofed Surface Plasmon Polariton (SSPP) waveguide which is basically an array of identical H-shaped SSPP unit cells formed by lower and upper corrugated edge of the antenna (See Fig. 1 for more details). The corrugated edges of the SSPP waveguide basically support propagation of slow traveling electromagnetic waves at more or less the same group velocity, which is less than the speed of light. Because of their being a slow traveling wave, these traveling waves are tightly bound to their respective corrugated edges to different extents.

In this proposed SSPP antenna topology, the period *p*′ and the grove width *g*′ are the same for both the upper and lower corrugated edges but the groove depths *d*′ of the upper corrugated edge is larger than that of the lower corrugated edge *de*′. The deeper grooves along the upper corrugated edges allow more electromagnetic energy to be stored along the upper corrugated edge. The tapered ground planes in the CPW feeding port has been designed to transform the input impedance from 50 ohm to the characteristic impedance of the guiding body gradually instead of abruptly. The presence of the tapered and elongated CPW ground planes absorbs the leaky modes and converts them into quasi-TEM modes in the CPW feeding port. The fundamental mode of the spoof surface plasmon transmission line is a slow-wave bounded mode that does not radiate due to the phase mismatch with the wave in the air. In order to make this kind of structure efficiently radiate power as an antenna, there are two feasible approaches, i.e., exciting the higher fast-wave modes of TLs or introducing deliberately periodic discontinuities/modulations in the guiding structure. A symmetrical profile in transversal or longitudinal plane usually suffers from a low radiation efficiency and makes it difficult to achieve exactly endfire radiations along with broadband characteristic. It suffers from the broadside or endfire radiation degradation issue, with a radiation efficiency theoretically limited to around 50 percent. This further leads to non-continuous beam steering in one particular direction (as reported in comparison table later). Here we introduce asymmetric periodic discontinuities in the proposed design which will overcome these issues in spoof surface plasmon based endfire radiating antennas. In the tapered end, the groove depths gradually decrease to zero. The gradual disappearance of the groove depths also means that the group velocity of the travelling wave increasingly reaches the speed of light. Along each of the corrugated edges, the traveling wave turns leaky as it passes through this tapered end and radiates out in all the directions to different extents. Since the upper corrugated edge has more stored electromagnetic energy, the amount of stored electromagnetic energy available for leaking out in the tapered end is more abundant along the upper edge than the lower edge and the endfire beam steered upwards. The tapered part and groove depths have been optimized carefully so that the design can achieve maximum gain and efficiency along with scanning feature.

The dispersion analysis of the proposed SSPP endfire antenna with double corrugated edges (both sides of the design), which is hereafter referred to as Antenna X is performed by using Eigen mode solver of 3D EM simulation software CST Studio Suite. The design of Antenna X is shown in Fig. 1, which also displays the unit element (H-shaped) extracted from the guiding body of the antenna. The dispersion analysis which follows is based on this unit element. The H-shaped unit element has a period (*p*′) of 4 mm, length (*h*′) of 9 mm, gap between to arms (*g*′) of 1 mm, groove depth (*d*′) of 4.0 mm from the top edge and groove depth dimension of (1 mm × 1 mm) from the bottom side. The total length (*L*′) of the antenna is 55 mm. The main body of the antenna is 40 mm in length (*La*′). The width of the CPW feeding section is 30 mm in width (*W*′). The triangular ground planes in the CPW feeding section has been optimized to minimize the sidelobe radiations.

**Figure 2:**
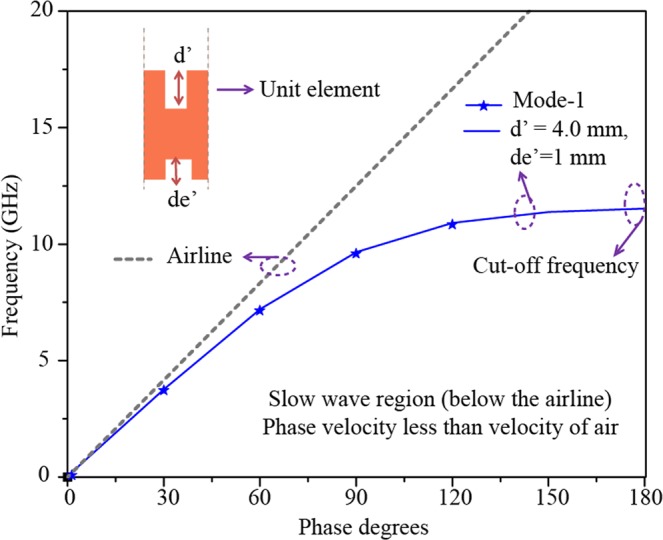
Dispersion graph of the unit cell extracted from the guiding body of SSPP antenna X.

The main body of the antenna is terminated with a tapered end to provide a wide impedance matching. Due to the presence of the tapered end, the effective aperture *a*′ is further reduced to just 3 mm, which is even less than a quarter of free space wavelength. The tapered part is 17 mm long and 3 mm wide. The whole antenna is realized on a Rogers Duroid 5880 substrate of thickness 0.8 mm and dielectric constant 2.2. The dispersion diagram for the proposed unit element of Antenna X is shown in Fig. 2. Periodic boundary conditions have been applied to the unit element in the direction of propagation of the wave along x-axis. The dominant mode lies just below the airline. Changing the groove depths can change the dispersion characteristics and the cutoff frequencies. According to our simulation results, an array of unit cells of an upper groove depth of 4.0 mm and a lower groove depth of 1 mm was found to satisfy the Hansen-Woodyard condition^23^ required for the endfire radiation with a desired performance. For endfire radiation the operating frequency in the dispersion graph should be below the airline in the slow wave region. By projection using the dispersion graph as shown in Fig. 2, the upper cut off frequency was found to be around 12 GHz.

**Figure 3:**
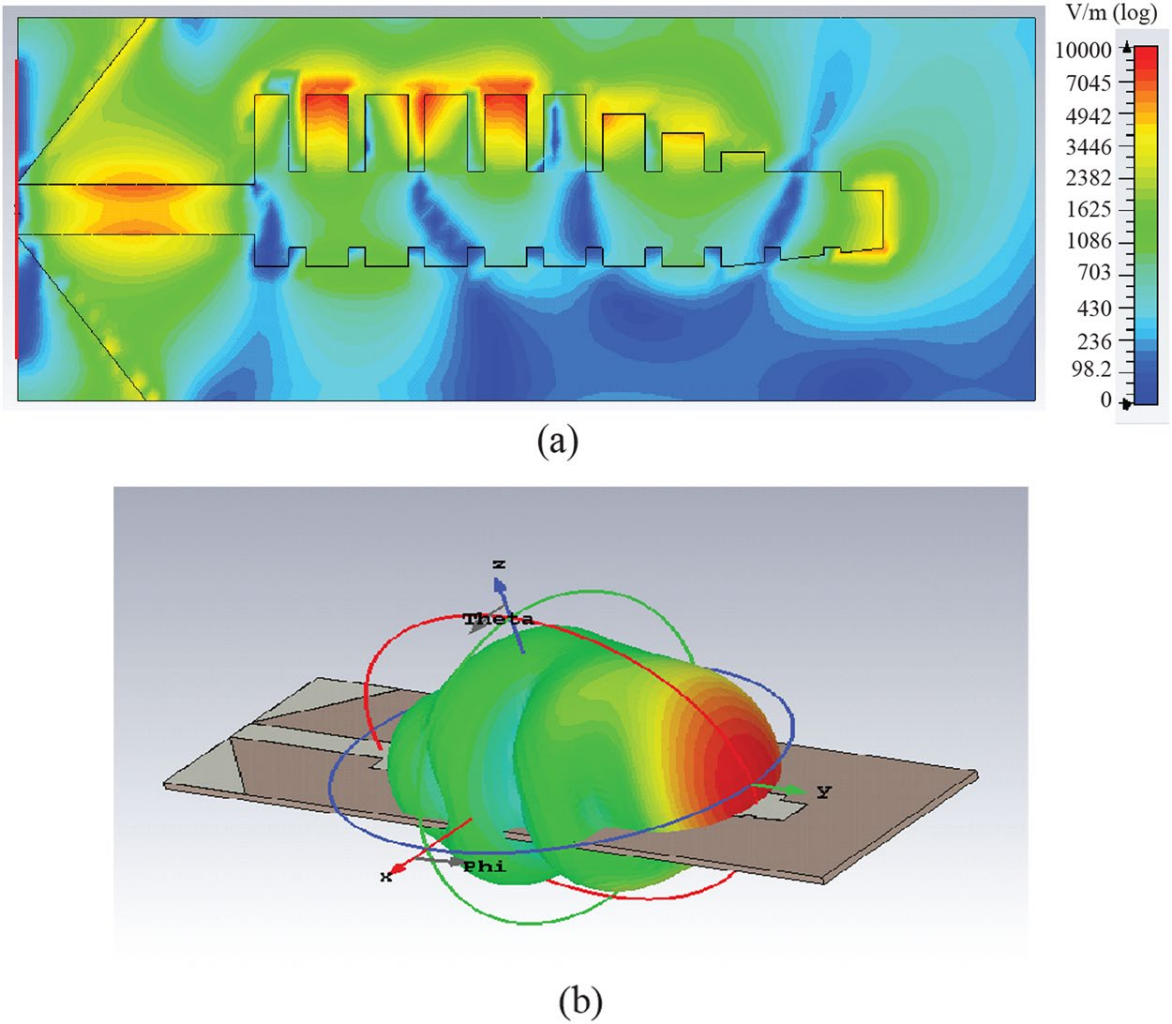
(**a**) Electric field distribution of SSPP antenna X at 10.5 GHz. (**b**) 3-D radiation pattern of SSPP antenna X at 10.5 GHz.

Figure 3a shows the field distribution of a traveling wave in Antenna X at 10.5 GHz. The results of simulation suggest that the travelling has propagated towards the positive x-direction with the electric field highly confined to both corrugated edges until it reaches the tapered end, where it finally radiates in the exact endfire direction. Figure 3b shows the 3D plot of the radiation of Antenna X in dBi scale. The data as shown in Fig. 3b is consistent with the electric field distribution as shown in Fig. 3a.

**Figure 4:**
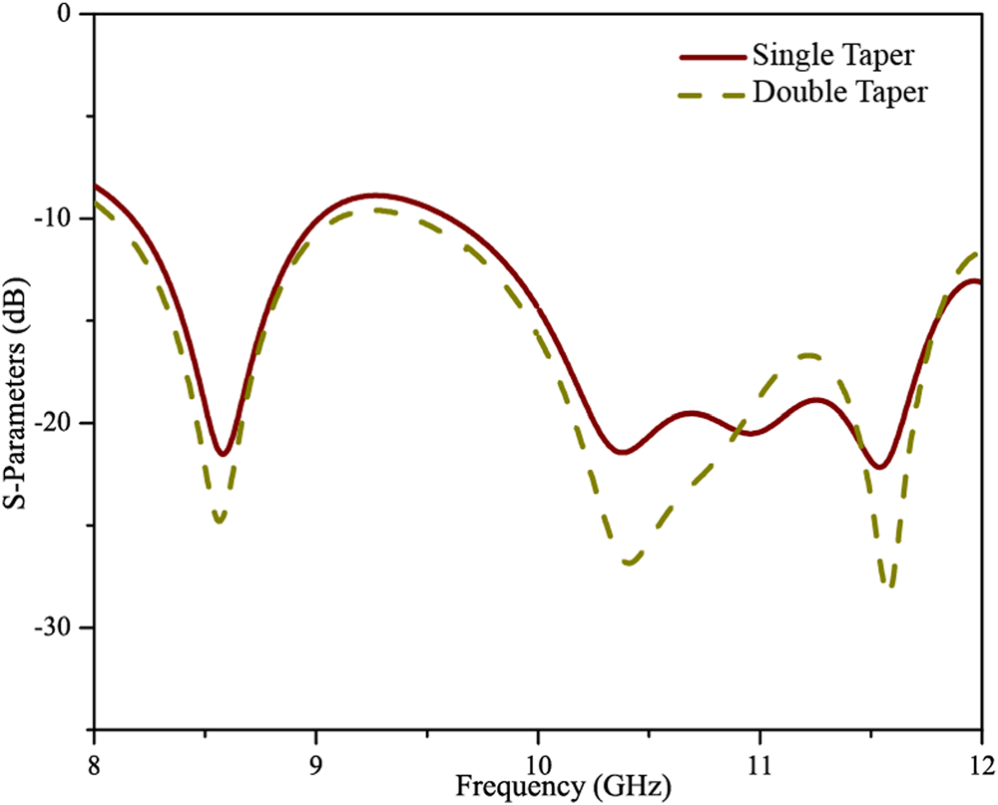
Scattering Parameters of SSPP antenna X.

Figure 4 shows the simulated scattering parameter (S-parameter) graph for Antenna X. The graph shows that a return loss of around 25 dB has been obtained in a wide operating frequency band. A wideband impedance matching has been achieved from 8 GHz to 12 GHz. Figure 4 contains two S-parameter plots: one for Antenna X with a single tapered end (one side of design) and other for Antenna X with double tapered ends (both sides tapered). As can be clearly seen, Antenna X with double tapered ends has yielded an improvement in S11 parameters. The return loss has been improved by 6 dB. The double tapered edges in Antenna X have been found to give a better impedance matching with the free space and hence to suppress the reflection at the end of the antenna. The tapered length has optimized to minimize the reflection without sacrificing the overall gain. With inclusion of the tapered end with tapered at both upper and lower edges, the reflections have been suppressed to less than −10 dB within a wide frequency band from 8 GHz to 12 GHz.

## Experimental Validations

**Figure 5:**
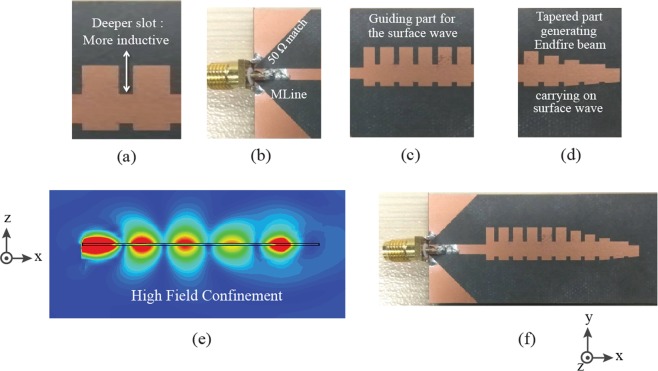
Proposed SSPP antenna X (**a**) Unit element (**b**) Transition part (**c**) Guiding part of the SSPP (**d**) Tapered part of the SSPP (**e**) Fields along the axis cut plane (**f**) Final Prototype.

The fabricated SSPP antenna X is shown in Fig. 5. Figure 5(a–d) shows the different parts of the fabricated design explaining their functions. Figure 5(e) shows the high field confinement essential for achieving endfire radiations. Figure 5(f) shows the final fabricated prototype design. The reflection response has been measured using an vector network analyzer (Agilent Technologies, Model No. 8753A), and the radiation patterns using an anechoic chamber.

**Figure 6:**
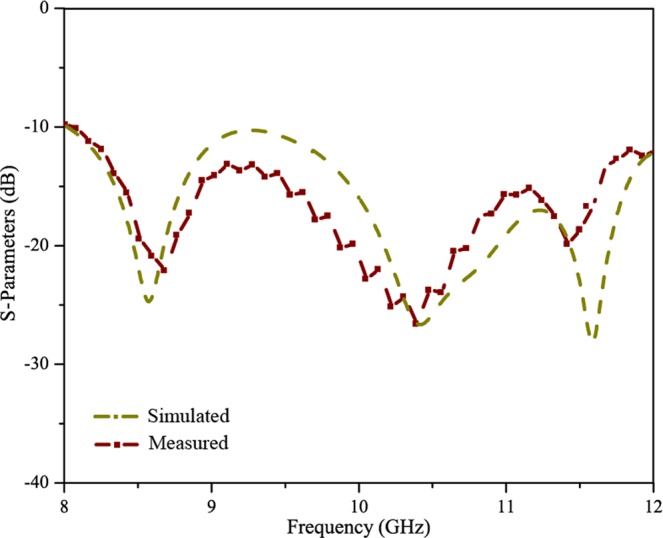
Measured scattering parameters of SSPP antenna X.

**Figure 7:**
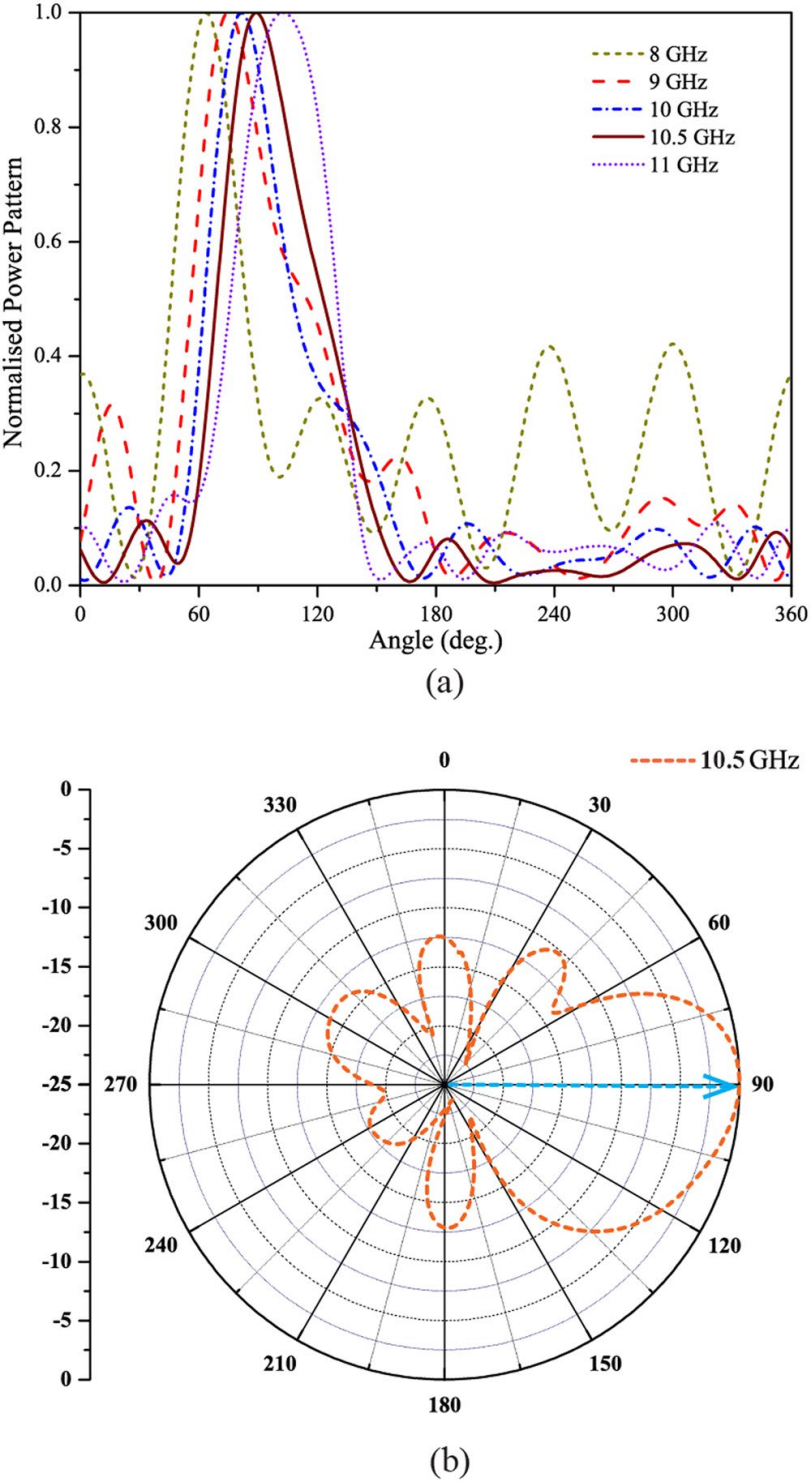
(**a**) Measured scanning patterns of SSPP antenna X. (**b**) Measured endfire radiation pattern of SSPP antenna X.

Figure 6 shows the measured scattering parameters for Antenna X. As can be seen from Fig. 6, both the measured results agree well with the simulated results as shown in Fig. 4. The measured scattering parameters are suppressed to less than −10 dB within 8 GHz-12 GHz for Antenna X. From Fig. 6, an impedance matching over a larger bandwidth has been achieved using Antenna X. Figure 7a shows the measured normalised radiation patterns of Antenna X as a function of beam angle. This plot displays a clear picture of the scanning range and the beam directions at various frequencies from 8 GHz to 12 GHz. At 8 GHz, the beam direction is 64 deg. The side lobe levels are slightly higher at this frequency because of greater reflections at the edge of the operating frequency band. The beam angles at frequencies 9 GHz, 10 GHz, 10.5 GHz and 11 GHz are respectively 75 deg., 82 deg., 90 deg. and 110 deg. Obviously, antenna has achieved a broadband impedance bandwidth and a correspondingly large smooth continuous scanning in the operating frequency band. The minimum side lobe levels are around −10 dB at all frequencies. As the antenna has a broad operating band, the frequencies other than the one required for exactly endfire radiation, can be harnessed for scanning applications. Figure 7b shows the measured normalised radiation pattern of Antenna X in endfire direction. The radiation pattern has been plotted for frequency of 10.5 GHz, where the exact endfire radiation has occurred. The maximum gain obtained at this frequency is about 9.5 dBi along with side lobe levels of about −10 dB.

**Figure 8:**
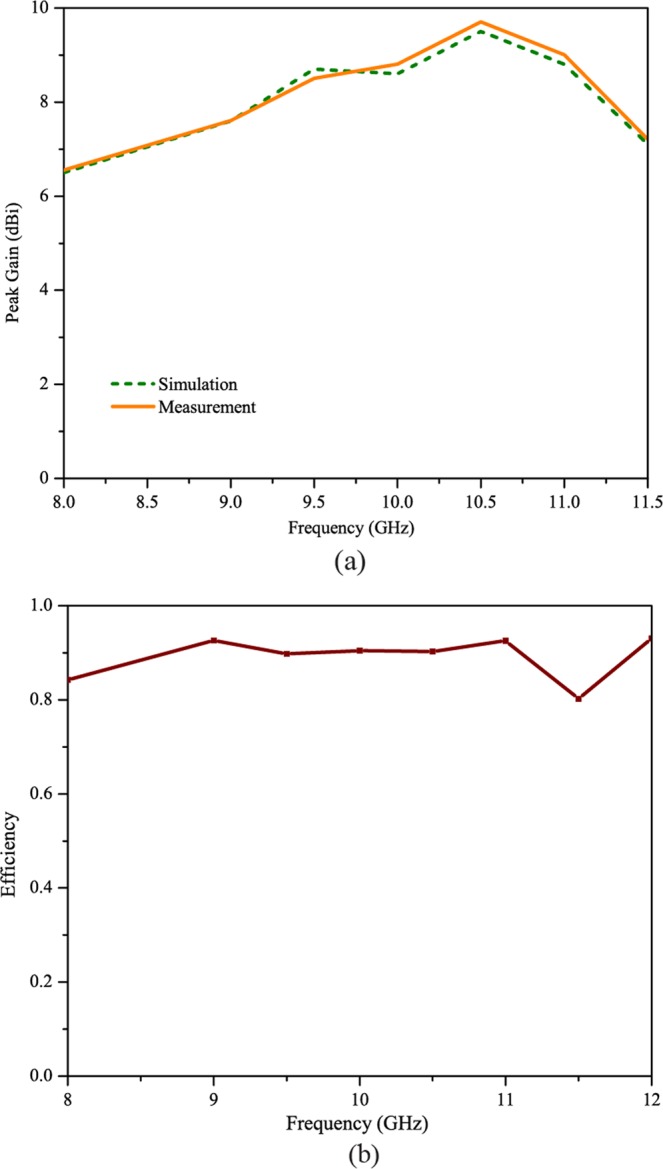
(**a**) Measured and simulated peak gain of SSPP antenna X. (**b**) Efficiency of the proposed SSPP antenna X.

Figure 8a shows the simulated and measured peak gain of Antenna X. The maximum simulated gain of antenna was around 9.6 dB, whilst the measured gain was even slightly higher i.e. 9.8 dBi. The graph trend shows that the maximum gain and radiations have been achieved at the exact endfire direction, due to the fact that, at this direction, the field was confined along the guiding body of antenna and most of the electromagnetic energy ends up being “squeezed” at the exact endfire direction. Figure 8b shows the measured efficiencies for Antenna X. The efficiency varies from 82 percent to 95 percent across the whole X-band. The maximum efficiencies obtained for the proposed antenna is about 95 percent.

## Application for Target Detection

This section propose a concept-of-proof of target detection application using proposed design as a multifunction. Microwave imaging is very useful for applications such has target detection. In order to enhance the cross-range resolution of the imaging system, many antennas can be distributed over an area within spacing less than the operating wavelength. But the mutual coupling between different antennas will be more if they are placed close to each other. This will in turn degrade the accuracy of the received signals. To solve these issues, one single antenna is required instead of many antennas. This kind of setup can scan over a large sampling area and the collected data can be mapped together. Thus we require an antenna that can scan at some angles to mitigate the requirement of multiple antennas. For this purpose the scanning antenna is very beneficial that can locate the target at some angle.

**Figure 9:**
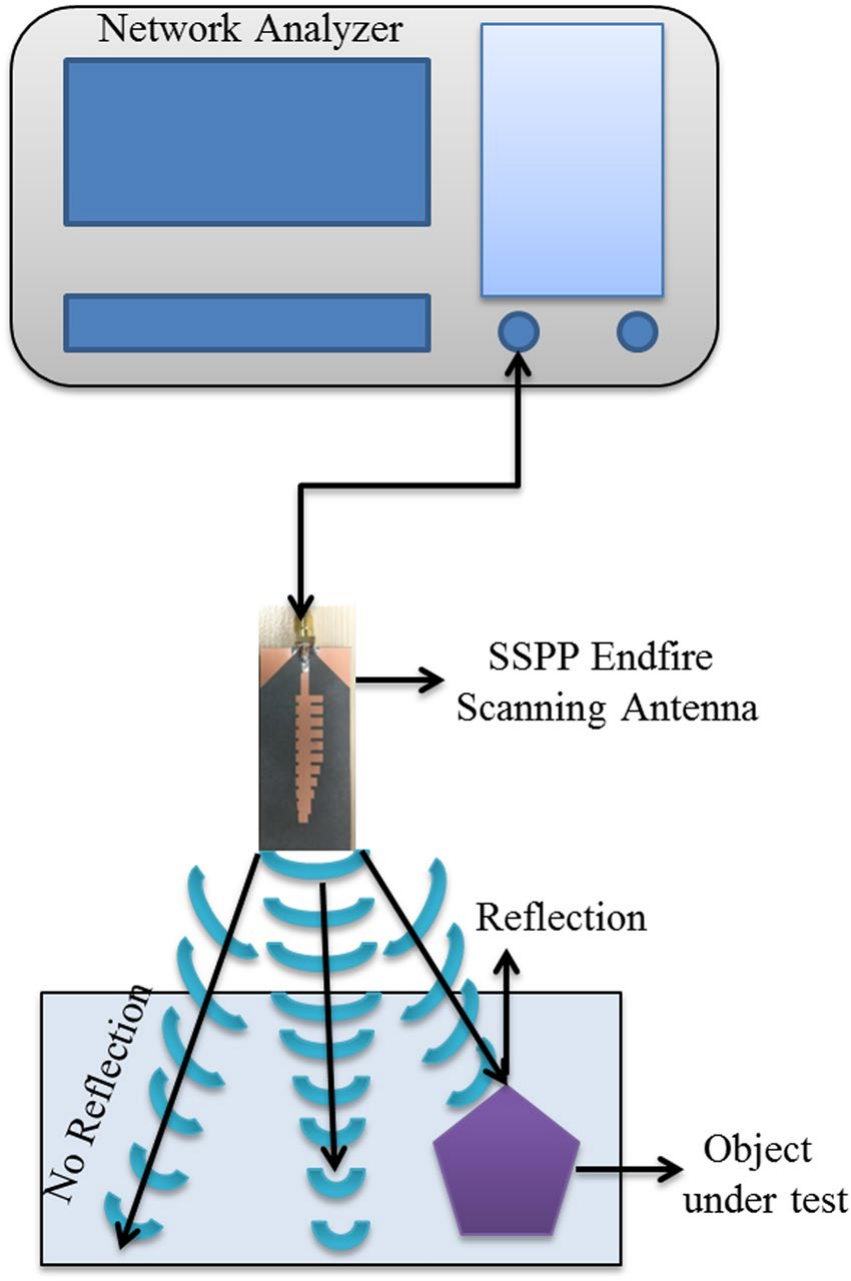
Measurement setup for the target location using SSPP antenna X.

The target detection system proposed in this paper is composed of a spoof surface plasmon antenna, object under test, measurement instrument and simulation software as shown in Fig. 9. The measurement instrument collects data from the sample under test. The proposed SSPP scanning transmitting antenna sends electromagnetic waves towards the direction of the sample under test. If the sample is made of only homogeneous material then there will be no wave reflected back. In case if there is any object placed in the direction of wave propagation, then it will reflect a portion of the electromagnetic wave. The more is the difference between the properties of the object under test and the surrounding medium, the stronger will be the reflection.

**Figure 10:**
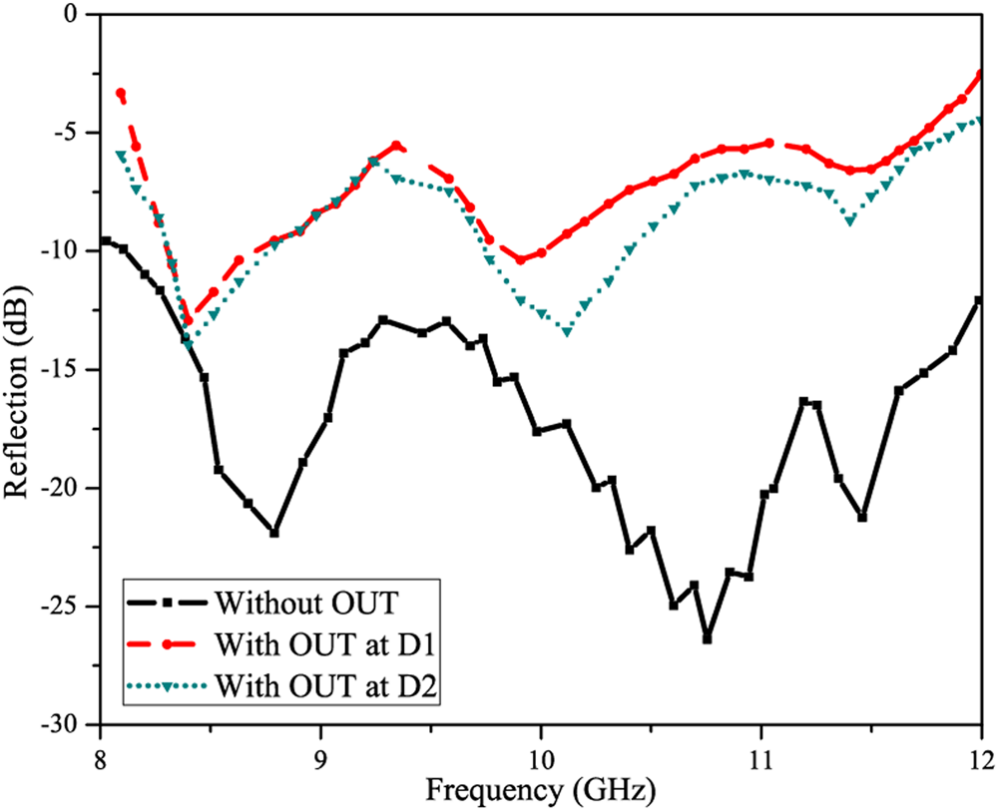
Reflection measurements at different distance.

Figure 10 shows the reflection parameters for the proposed detection system. The object under test (OUT) has been placed at two different distances (D1 and D2) and the reflection has been measured. It can be clearly seen that the reflection changes when the object is placed in the way. In order to show the benefit of scanning antenna, the object has been slightly placed off-straight path. As the proposed SSPP antenna is scanning at wide scanning range, the OUT can be located by observing the change in the reflection. The reflection is much higher when the object is placed at some angle at some distance within the scanning range. The reflection is more at distance D1 (5 cm) and slightly less at distance D2 (10 cm) which proves that the distance also plays a role in the detection measurements.

**Figure 11:**
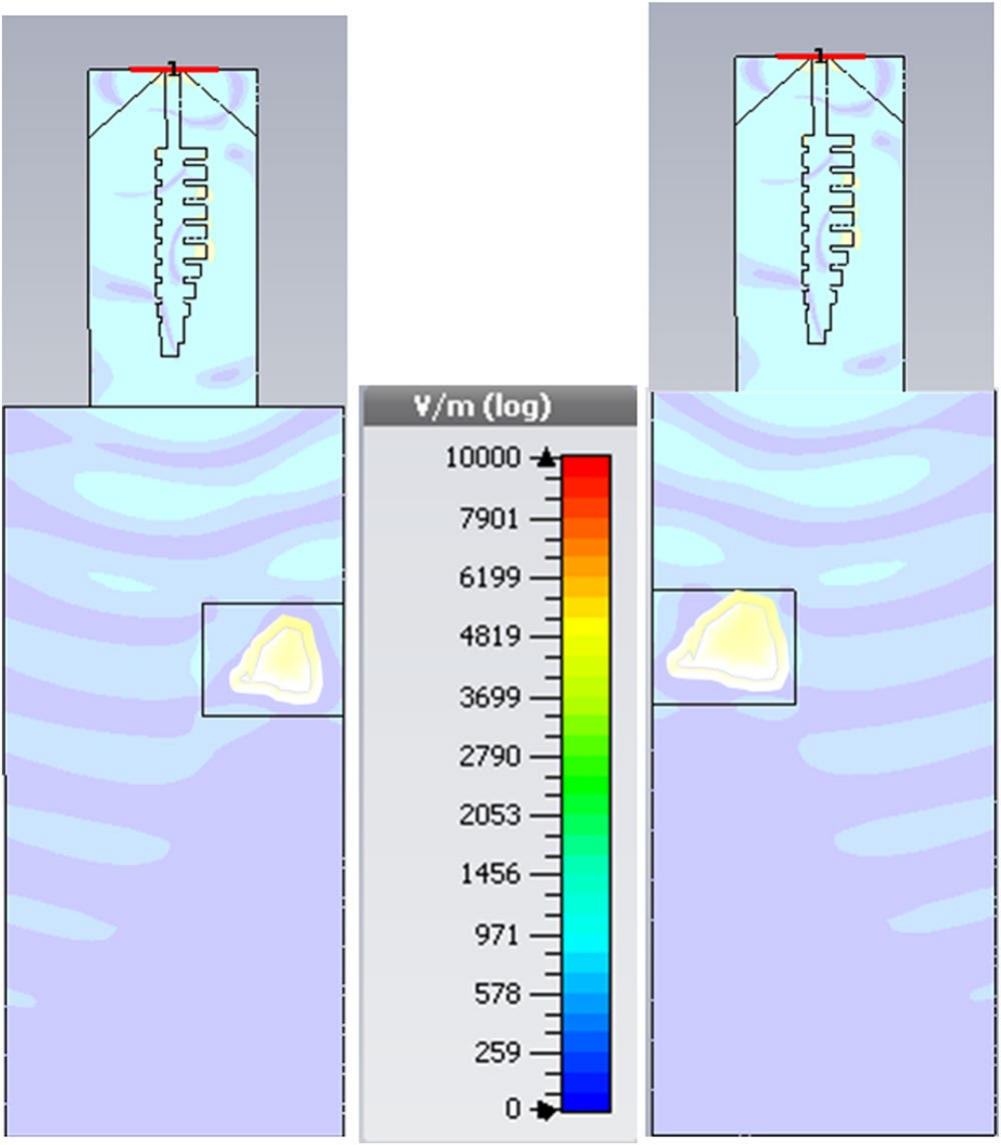
Field distribution for target at different locations.

Figure 11 shows the field distribution graphs showing the wave propagation when the target is placed at different locations. Case-I shows that when the target is placed at the right side within the scanning range of the SSPP antenna, the reflection can be clearly observed. The wave stops travelling in the direction of target due to reflections. Similarly for Case-II, due to the reflections the field is distributed only in one side within the scanning range.

This concept-of-proof experiment shows that the proposed kind of SSP based designs can be beneficial for target detection or other frequency scanning applications. Although the side lobe levels are not as low as required for imaging but the design has provided good results in terms of high efficiency and gain. However the proposed methodology can be further improved in the future work.

Table 1 shows the comparison of published spoof surface plasmon based endfire radiating antennas with our proposed design. Here ‘OS’ is the overall size, ‘BW’ is the bandwidth, ‘EG’ is the endfire gain and ‘NL’ is non-linear. In^18,19^, high gain is obtained at the expense of large dimensions however the bandwidth is still lower than our proposed design. In^19^, endfire scanning has been observed but non-linearly and the scanning range is very less. Therefore, it can be clearly observed from the table that our proposed design is very compact as compared to other SSPP endfire antenna designs. Also the bandwidth achieved is quite large with a high gain. The extra feature of scanning along endfire direction is also achieved with large scanning range. Beam scanning in broadband SSPP endfire radiating antenna is a relatively unexplored area of research. The scanning feature of proposed SSPP antenna can be used as a multifunction in frequency scanning applications. One such application has been tested experimentally to prove the scanning effect for target location application in particular.

**Table 1:** Comparison with the SSPP antenna counterparts.

Reference	Dimensions(Wavelength)	Frequency (GHz)	BW(GHz)	EG(dBi)	Endfire scan
^13^	2.4 *λ* × 0.66 *λ*	6	0.2	7.0	No
^11^	3.33 *λ* × 0.74 *λ*	5.5	0.5	7.8	No
^15^	3.65 *λ* × 0.8 *λ*	5.0	0.5	9.0	Yes (4 deg.)
^18^	5.76 *λ* × 2.1 *λ*	4.75	3.5	13.3	No
^19^	5.5 *λ* × 0.33 *λ*	5.0	1.0	12.9	Yes (NL)
Our work	1.8 *λ* × 1.0 *λ*	10.0	4.0	9.8	Yes (35 deg.)

## Conclusions

A low-profile and efficient SSPP antenna has been successfully realized for endfire radiations and frequency scanning applications. Antenna designed has been found to be able to provide through endfire scanning in X-band. The effective aperture of the antenna has been reduced to less than half wavelength, the peak efficiency is in excess of 95 percent and a peak gain in excess of 9.8 dBi. As a concept-of-proof, the SSP antenna has also been tested for target detection, location application using reflection method and the experiments show promising results. It can be further extended on system level with the improved characteristics. The proposed multi-function design can be used for other applications where scanning antennas are required.

## References

[1] Maier, S. A. *Plasmonics: Fundamentals and Applications* (Springer, New York, 2007).

[2] Barnes WL, Dereux A, Ebbesen TW (2003). Surface plasmon subwavelength optics. Nature.

[3] Pendry JB, Martin-Moreno L, Garcia-Vidal FJ (2004). Surface plasmon subwavelength optics. Science.

[4] Gibson, P. J. The vivaldi aerial. *9th European Microwave Conference Proceedings* 101–105 (1979).

[5] Gross, F. *Frontiers in Antennas: Next Generation Design and Engineering* (The McGraw-Hill Companies, 2011).

[6] Tang HH, Ma TJ, Liu PK (2016). Experimental demonstration of ultra-wideband and high-efficiency terahertz spoof surface plasmon polaritons coupler. Appl. Phys. Lett..

[7] Shen XP, Cui TJ (2013). Planar plasmonic metamaterial on a thin film with nearly zero thickness. Appl. Phys. Lett..

[8] Guan D, You P, Zhang Q, Xiao K, Yong S (2017). Hybrid spoof surface plasmon polariton and substrate integrated waveguide transmission line and its application in filter. IEEE Transactions on Microw. Theory Tech..

[9] Kianinejad A, Chen ZN, Qiu C (2016). Low-loss spoof surface plasmon slow-wave transmission lines with compact transition and high isolation. IEEE Transactions on Microw. Theory Tech..

[10] Liu X (2014). Planar surface plasmonic waveguide devices based on symmetric corrugated thin film structures. Opt. Express.

[11] Liu X, Feng Y, Zhu B, Zhao J, Jiang T (2013). High-order modes of spoof surface plasmonic wave transmission on thin metal film structure. Opt. Express.

[12] Li Z (2016). Coplanar waveguide wideband band-stop filter based on localized spoof surface plasmons. Appl. Opt..

[13] Yin JY (2017). Endfire radiations of spoof surface plasmon polaritons. IEEE Antennas and Wirel. Propag. Lett..

[14] Tian D (2018). Low-profile high-efficiency bidirectional endfire antenna based on spoof surface plasmon polaritons. IEEE Antennas and Wirel. Propag. Lett..

[15] Zhuang K (2019). Pattern reconfigurable antenna applying spoof surface plasmon polaritons for wide angle beam steering. IEEE Access.

[16] Xu JJ, Zhang HC, Zhang Q, Cui TJ (2015). Efficient conversion of surface-plasmon-like modes to spatial radiated modes. Appl. Phys. Lett..

[17] Chopra, R. & Kumar, G. Uniplanar microstrip antenna for endfire radiation. *IEEE Transactions on Antennas and Propagation* 1–1 (2019).

[18] Hou Y, Li Y, Zhang Z, Iskander MF (2019). Microstrip-fed surface-wave antenna for endfire radiation. IEEE Transactions on Antennas Propag..

[19] Hou Y, Li Y, Zhang Z, Feng Z (2018). Narrow width periodic leaky-wave antenna array for endfire radiation based on hansen woodyard condition. IEEE Transactions on Antennas Propag..

[20] Yin JY, Zhang HC, Fan Y, Cui TJ (2016). Direct radiations of surface plasmon polariton waves by gradient groove depth and flaring metal structure. IEEE Antennas Wirel. Propag. Lett..

[21] Tang, X.-L. *et al*. Continuous beam steering through broadside using asymmetrically modulated goubau line leaky-wave antennas. *Sci. Rep*. **7** (2017). 10.1038/s41598-017-12118-8 PMC5601911 28916819

[22] Kandwal A, Zhang Q, Tang X, Liu LW, Zhang G (2018). Low-profile spoof surface plasmon polaritons traveling-wave antenna for near-endfire radiation. IEEE Antennas and Wirel. Propag. Lett..

[23] Hansen, W. W. Radiating electromagnetic wave guide, in google patent 2 402 622. *Wiley* (1946).

